# Can community structure track sea‐level rise? Stress and competitive controls in tidal wetlands

**DOI:** 10.1002/ece3.2758

**Published:** 2017-01-27

**Authors:** Lisa M. Schile, John C. Callaway, Katharine N. Suding, N. Maggi Kelly

**Affiliations:** ^1^ Department of Environmental Science, Policy, and ManagementUniversity of CaliforniaBerkeleyCAUSA; ^2^ Department of Environmental ScienceUniversity of San FranciscoSan FranciscoCAUSA; ^3^ Present address: Smithsonian Environmental Research Center647 Contees Wharf Rd.EdgewaterMD21037USA; ^4^ Present address: Department of Ecology and Evolutionary BiologyUniversity of ColoradoRamaley N122Campus Box 334BoulderCO80309USA

**Keywords:** competition, facilitation, *Schoenoplectus acutus*, *Schoenoplectus americanus*, sea‐level rise, tidal wetlands

## Abstract

Climate change impacts, such as accelerated sea‐level rise, will affect stress gradients, yet impacts on competition/stress tolerance trade‐offs and shifts in distributions are unclear. Ecosystems with strong stress gradients, such as estuaries, allow for space‐for‐time substitutions of stress factors and can give insight into future climate‐related shifts in both resource and nonresource stresses. We tested the stress gradient hypothesis and examined the effect of increased inundation stress and biotic interactions on growth and survival of two congeneric wetland sedges, *Schoenoplectus acutus* and *Schoenoplectus americanus*. We simulated sea‐level rise across existing marsh elevations and those not currently found to reflect potential future sea‐level rise conditions in two tidal wetlands differing in salinity. Plants were grown individually and together at five tidal elevations, the lowest simulating an 80‐cm increase in sea level, and harvested to assess differences in biomass after one growing season. Inundation time, salinity, sulfides, and redox potential were measured concurrently. As predicted, increasing inundation reduced biomass of the species commonly found at higher marsh elevations, with little effect on the species found along channel margins. The presence of neighbors reduced total biomass of both species, particularly at the highest elevation; facilitation did not occur at any elevation. Contrary to predictions, we documented the competitive superiority of the stress tolerator under increased inundation, which was not predicted by the stress gradient hypothesis. Multifactor manipulation experiments addressing plant response to accelerated climate change are integral to creating a more realistic, valuable, and needed assessment of potential ecosystem response. Our results point to the important and unpredicted synergies between physical stressors, which are predicted to increase in intensity with climate change, and competitive forces on biomass as stresses increase.

## 1 Introduction

Climate change will influence plant communities through shifts in temperature, carbon dioxide concentrations, precipitation, nitrogen, and sea level, among other abiotic factors, and shifts are apparent already in plant distribution, productivity, and phenology (Dieleman et al., 2012; Garcia, Cabeza, Rahbek, & Araújo, 2014; Jump & Peñuelas, 2005; Parmesan & Yohe, 2003; Sproull, Quigley, Sher, & González, 2015; Zavaleta, Shaw, Chiariello, Mooney, & Field, 2003). In tidal wetlands, the critical abiotic factors affecting plant distributions are anaerobic conditions created through inundation duration and depth and salinity (Howard, Biagas, & Allain, 2016; McKee, Cahoon, & Feller, 2007; McKee & Mendelssohn, 1989; Mendelssohn, McKee, & Patrick, 1981), and these factors are likely to be highly affected by climate change (Kirwan & Megonigal, 2013). Biotic factors also can affect plant distributions through competition by directly excluding or reducing performance (Crain, Silliman, Bertness, & Bertness, 2004; Emery, Ewanchuk, & Bertness, 2001; Grace & Wetzel, 1981) or through facilitation, via amelioration of salinity stress (shading) or anaerobic stress (soil aeration by both plants and animals; see review in Zhang and Shao (2013)). While the impact and interactions of abiotic and biotic stresses are likely to shift with climate change (Brooker, 1996; Suttle, Thomsen, & Power, 2007), little is known about the role of accelerated climate change in the context of trade‐offs among stress tolerance, competition, and facilitation (Adler, Dalgleish, & Ellner, 2012; Gilman, Urban, Tewksbury, Gilchrist, & Holt, 2010; Maestre et al., 2010).

The framework of the stress gradient hypothesis (SGH) is applicable in addressing these future climate change impacts. The SGH posits that biotic interactions are driven by facilitation under conditions of high abiotic stresses, such as temperature, water availability, or inundation, and that competition drives interactions under more benign conditions (Bertness & Callaway, 1994; Maestre, Callaway, Valladares, & Lortie, 2009). A meta‐analysis of plant species interactions by He, Bertness, and Altieri (2013) identified a high occurrence of facilitation or a reduction in competition with increasing stress, suggesting that facilitation might play a larger role in species interactions with accelerated climate change. In addition, the physiological status of a plant can affect morphology (Schöb, Armas, Guler, Prieto, & Pugnaire, 2013) as well as life stage (Engels, Rink, & Jensen, 2011), which in turn can vary facilitative effects. Yet, many uncertainties remain regarding how species distribution and abundance will be affected, and how the nature (resource vs. nonresource stress) and severity of the stress will affect interactions (He et al., 2013).

Ecosystems with strong stress gradients, such as mountain slopes, estuaries, and the rocky intertidal, allow for space‐for‐time substitutions of stress factors and can give insight into future climate‐related shifts in both resource and nonresource stresses. In particular, tidal wetlands are an ideal ecosystem to study the effect of climate change on species interactions due to the clear identification of dominant stressors (Crain et al., 2004; Pennings & Callaway, 1992), the compact nature of the gradient, and the significant negative effects of predicted climate change (Donnelly & Bertness, 2001). Sea levels are predicted to rise between 0.4 and 1.8 m by 2100 (Horton, Rahmstorf, Engelhart, & Kemp, 2014; Moore, Grinsted, Zwinger, & Jevrejeva, 2013; Vermeer & Rahmstorf, 2009), and concurrent with this rise are increases in estuarine salinity (Cloern et al., 2011). Although increases in sea‐level rise (SLR) may be counterbalanced by sediment accretion and increased belowground biomass production (Cherry, McKee, & Grace, 2009; Morris, Sundareshwar, Nietch, Kjerfve, & Cahoon, 2002; Schile et al., 2014), tidal wetlands are likely to lose relative elevation and experience increased rates of tidal inundation, leading to increased anaerobic stress (Chapman, 1977; Ungar, 1991), as well as shifts in the salinity gradient. Estuary‐level decreases in biomass are likely to occur because of increased salinity, and previous work has documented decreases in site‐level biomass with increased salinity in brackish marshes (Craft et al., 2008; Crain et al., 2004; Neubauer & Craft, 2009). Both competitive (Pennings & Callaway, 1992) and facilitative interactions (Bertness & Callaway, 1994; Bertness & Hacker, 1994) have been documented within wetlands. Specifically with facilitation, inundation‐tolerant species possess a high proportion of aerenchymatous tissue, which increases oxygen flow to belowground organs and subsequently can oxygenate soil, increase soil redox potential, and enable growth of species less tolerant of anoxic conditions (Hacker & Bertness 1995; Kludze & DeLaune, 1995; Callaway & King, 1996a,b; Jackson & Armstrong, 1999). Examining whether these processes can occur within this relatively simple system could give insight into similar dynamics in other ecosystems with strong stress gradients such as chaparral, deserts, and the rocky intertidal.

In this paper, we test the effect of abiotic stress, specifically inundation stress, and biotic interactions (facilitation and competition) on plant growth and survival under field conditions using experimental planters called “marsh organs” (Morris, 2007), which allow for the manipulation of elevation to simulate SLR across existing marsh elevations and those not currently found within marshes to reflect potential future conditions (Kirwan & Guntenspergen, 2012; Langley, Mozdzer, Shepard, Hagerty, & Patrick Megonigal, 2013; Voss, Christian, & Morris, 2013). We define stress simplistically as a reduction in biomass (Grime, 1979). We chose two cosmopolitan wetland sedge species, one dominant at low elevations, *Schoenoplectus acutus*, and one dominant at marsh plain elevations, *Schoenoplectus americanus*, that have adjacent, slightly overlapping tidal distributions in the San Francisco Bay estuary, California, USA. Over one growing season, we investigated the individual and combined effects of increased inundation and biotic interactions on above‐ and belowground biomass of these species at two tidal brackish wetlands that differ slightly in salinity. Based on current marsh distributions, we hypothesized that: (1) without competition, *S. acutus* would perform better than its congener, *S. americanus*, under increased inundation stress; (2) *S. americanus* would have a competitive advantage under conditions of lower inundation stress; and (3) when grown together, *S. acutus* would facilitate the growth of its congener under the greatest inundation stress (increased facilitation via the alleviation of anaerobic conditions) owing to its potential to aerate anoxic soil through its rich aerenchymatous tissue (Sloey, Howard, & Hester, 2016).

## 2 Materials and Methods

### 2.1 Site description

We conducted the experiment within two historic brackish tidal wetlands: Browns Island (latitude: 38°2′16″N, longitude: 121°51′50″W) and Rush Ranch Open Space Preserve (latitude: 38°11′48″N, longitude: 122°01′44″W; Fig. S1). Both sites experience mixed semidiurnal tides. Water salinity fluctuates seasonally, with the lowest and highest salinities found in the early spring and early fall, respectively, and the magnitude depends on winter precipitation, snow pack, and river flow (Fig. S2; Enright & Culberson, 2009). The average water salinity between 2008 and 2011 was 1.5 and 4.3‰ at Browns Island (“fresher site”) and Rush Ranch (“saltier site”), respectively, and salinity was consistently higher, although not markedly, at Rush Ranch throughout and across years (Fig. S2). The year that this study was conducted was not considered to be a drought year; therefore, channel water salinities were more similar between sites during most the experiment, but started to increase at the end of the experiment (Fig. S2). Although the difference in salinity is small, the effect on species diversity (Vasey et al. 2012) and biomass (Vasey, Parker, Herbert, & Schile unpublished data) is notable.

### 2.2 Species description

A common marsh plain species *S*. *americanus* (Pers.) Volkart ex Schinz & R. Keller (Olney's bulrush) forms solid stands across mid‐ and high marshes. Stems are 0.3–1.8 m tall, and rhizomes are 0.5–2 cm wide, forming both clumps and runners (Ikegami, Whigam, & Werger, 2007). *Schoenoplectus americanus* has been studied widely under a variety of climate change and competition scenarios along the Atlantic coast and Gulf of Mexico, including flooding, increased carbon dioxide concentrations, and nutrient addition (Broome, Mendelssohn, & McKee, 1995; Erickson, Megonigal, Peresta, & Drake, 2007; Kirwan & Guntenspergen, 2012; Langley & Megonigal, 2010; Langley et al., 2013); however, field experiments have not specifically addressed how SLR affects abiotic and biotic interactions. Dominating in the low marsh, *S. acutus* (Muhl. ex Bigelow) Á. Löve & D. Löve var. *occidentalis* (S. Watson) S.G. Sm. (hardstem tule) grows along tidal channel, river, and lake margins and forms stands of erect 1.5–3‐m‐tall stems. Rhizomes are 1.5–4 cm wide and grow linearly with few branches (Wildová, Gough, Herben, Hershock, & Goldberg, 2007). Little is known about the responses of *S. acutus* to increased inundation and neighbor interactions in tidal systems; however, its ability to tolerate increased inundation rates has been documented (Sloey, Willis, & Hester, 2015; Sloey et al., 2016). Both species reproduce both clonally and through seeds; the frequency of either depends on environmental conditions (Ikegami, 2004).

### 2.3 Experimental design

**Figure 1:**
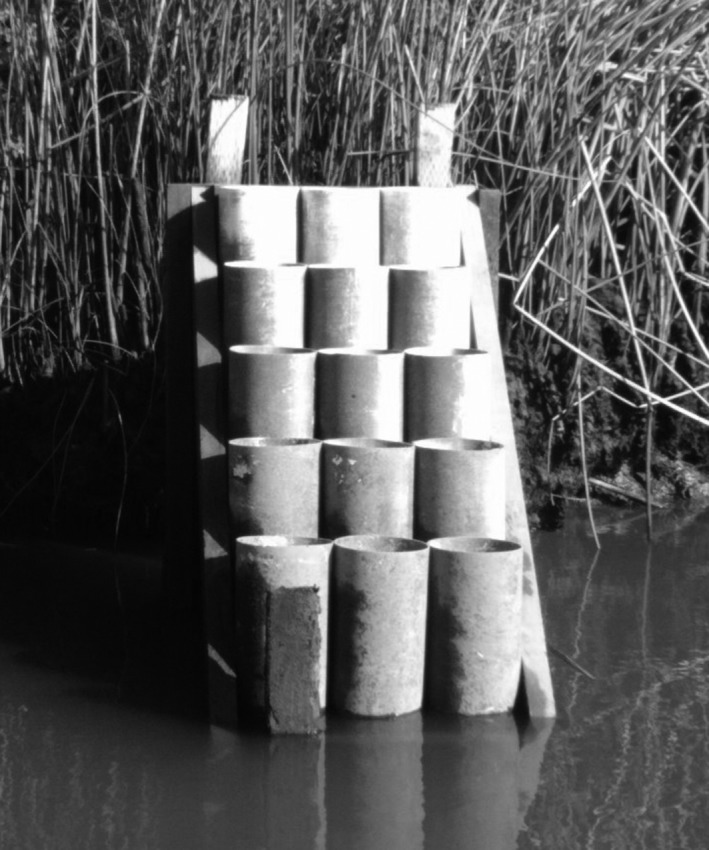
Unplanted marsh organ during low tide

Fourteen experimental planters (hereafter called marsh organs (Morris, 2007)) were constructed to grow both species in tidal channels at five fixed elevations that extend to approximately 80 cm lower than current vegetated marsh elevations (Figure 1); seven marsh organs were installed at a range of locations across each site. To avoid nonindependence of replicates within organs, we opted to build smaller organs with one replicate treatment per elevation and increase the number of organs per site rather than the more usual approach of constructing marsh organs with replicate treatments per elevation but using few organs per site. To construct a marsh organ, 15.2‐cm‐diameter PVC pipes were cut in triplicate to lengths of 45, 60, 75, 90, and 105 cm and each pipe bottom was covered in window screen mesh. In order of descending height, pipes were bolted together in rows of three by height class to form a flat‐bottomed structure, and pipes were bolted into a wood frame (Figure 1). At each wetland, seven south‐facing locations across multiple channels were chosen adjacent to the marsh edge, which was carried out to account for potential channel variability and minimize shading effects. Three support beams were pounded to resistance (~3.5 m) into the channel bottom, onto which the marsh organ was securely mounted. Using a Leica GPS1200 series real‐time kinematic global positioning system unit with vertical accuracy of 2–3 cm, the top row elevation was set at 1.5 ± 0.03 m NAVD88, which was determined based on surveys documenting the lower range of marsh elevations for *S. acutus* and *S. americanus*. Sediment to fill the pipes was collected from mudflats within each marsh, and additional sediment was added to the pipes for at least 1 month to compensate for compaction.

As noted in previous marsh organ experiments (Kirwan & Guntenspergen, 2012; Langley et al., 2013), this experimental design only allows for tidal drainage from the bottom of each tube and does not permit lateral flow. While this could amplify any potential inundation effects by increasing residence time, we do not feel that this effect was strong, if present, because no standing water was ever observed within a tube at low tide and tubes were observed to drain at a rate comparable to lowering tides. To account for potential restrictive effects of PVC size on belowground growth, we chose the largest available PVC tubes and ran the experiment for only one growing season.

### 2.4 Data collection

In April 2010, rhizomes of both species were collected from multiple locations within a 5 m diameter at the fresher site, washed, and grown in fresh water in a glasshouse. We chose to collect rhizomes from the fresher site to (1) control for maternal effects that could differ across site (although no data on genetic variability within sites have been collected within our literature review for either species); and (2) to use plants that predominantly experience freshwater conditions. No genetic analyses were conducted on the source material. In February 2011, all rhizomes and shoots were clipped to a standard length and weighed, and rhizomes were planted in the marsh organs at both sites in early March. Because we were focused on the effect of each species on the other, rather than comparing the relative importance of intra‐ and interspecific competition, we chose to use an additive design for our planting; one rhizome of each species was planted individually, and one rhizome of each species was planted together to examine the role of biotic interactions. Every month from April to September 2011, all stems were measured, and total stem length and stem density were calculated. Pore water salinity, pore water sulfides, and redox potential were collected monthly during low tides at both sites within the same week. Channel water level stations were installed at both sites and recorded water salinity and depth relative to meters NAVD88 every 15 min. The time inundated was calculated for each marsh organ elevation at both sites between March and September, and common tidal summaries (mean high water, mean low water, etc.) were computed. In one randomly selected pipe in every row of every organ, pore water was collected 15 cm deep. Salinity was measured, and 2–5 mL of pore water was mixed immediately with a sulfur antioxidant buffer solution in a vacuum‐evacuated vial. Sulfide concentrations were measured in the laboratory and compared against a standard curve. Every month at each wetland, one organ was randomly chosen to collect redox measurements, *Eh*, within every pipe. Platinum‐tipped redox electrodes were placed 15 cm deep, left for a day to equilibrate, and *Eh* was measured during the bottom of the low tide. *Eh* was calculated by adding the field voltage to a correction factor for the reference electrode (+200 mV). No pore water or *Eh* measurements were taken in August.

Aboveground biomass was removed between September 26 and 30 at the fresher site and October 10 and 13 at the saltier site; the difference in the timing of removal was due to high tides restricting access to all marsh organ elevations. All aboveground growth had stopped by the time of removal, and all biomass from a given organ was removed on the same day. Intact marsh organ tubes containing belowground biomass were removed between October 26 and 28 at Browns Island and October 31 and November 4 at Rush Ranch. Aboveground biomass was washed, sorted by species and live and dead shoots, dried at 70°C until a constant weight was obtained (typically 2 days), and weighed. Belowground biomass was removed from the pipes, washed thoroughly of all sediment over a 2‐mm screen, sorted by species, roots, and rhizomes, dried at 70°C until a constant weight (typically 3 days), and weighed.

### 2.5 Data analysis

All data were analyzed using SAS 9.2 (SAS, 2009); data transformations, when needed, are noted below, and all data met conditions of normality and homogeneity of variance. All post hoc comparisons were made using Tukey's least square means test. At both sites, the average number of minutes that each elevation treatment was inundated was analyzed using a two‐way analysis of variance (ANOVA). The data were log‐transformed. The effects of elevation and site on pore water salinity, sulfides, and *Eh* over time were analyzed using a repeated measures ANOVA (rmANOVA). Salinity and sulfides were square root transformed. A simple linear regression was run to test for effects of initial wet biomass on total harvested plant biomass. To address our first hypothesis at each site, differences in aboveground, belowground, total biomass, live‐to‐dead biomass ratio, and root‐to‐shoot ratio between species and among elevations were analyzed using a two‐way ANOVA, and all variables were square root transformed except for the live‐to‐dead biomass ratio, which was log‐transformed. We ran the same analysis with the same transformation to assess differences in the same biomass metrics of plants grown together. To address our second and third hypotheses, the natural log response ratio (lnRR; Suding, Goldberg, & Hartman, 2003) was calculated for each replicate row for each species:

$$\text{lnRR}=\text{ln}({\text{biomass}}_{\mathrm{with}\;\mathrm{neighbors}}/{\text{biomass}}_{\mathrm{without}\;\mathrm{neighbors}})$$

Values <0 indicate competition, whereas values >0 indicate facilitation. The lnRR was calculated for total biomass of both species within each organ row, and the treatment effects of elevation and site were analyzed using a one‐way *t*‐test (null expectation zero). Differences in lnRR among species at each elevation and site were analyzed using an ANOVA with planned comparisons.

## 3 Results

### 3.1 Abiotic measurements

Inundation duration increased significantly with decreasing elevation, and the effect differed by site (Fig. S3). The bottom three elevations at the fresher site were inundated longer than at the saltier site (*P *<* *0.004 for all comparisons); the top two elevations did not differ in inundation time between sites (*P *>* *0.90 for both comparisons). The depth of inundation was greater at the saltier site than at the fresher site by an average of 11 cm, and the tidal amplitude also was greater (0.72 m vs. 0.59 m; Table S1).

As expected, salinity stress was consistently higher at the saltier site than at the fresher site (Fig. S2); salinity increased over time, but only varied significantly among elevations in September (Table S2; Fig. S4). All *Eh* values were consistent with reduced, anaerobic conditions across all elevations and on average were lower at the saltier site (Table S2, Fig. S5). Both pore water sulfide concentrations and *Eh* varied over time, but there was no consistent or significant temporal trend or trend with elevation or species (Table S2; Fig. S5). Combining data across elevations, sulfide concentrations were higher at the saltier than at fresher site, but only marginally (*P* = 0.066).

### 3.2 Effects of initial biomass on total harvested biomass

There was no significant relationship between the initial biomass and total harvested biomass for *S. americanus* (data not shown; fresher site: *F*_1,68_ = 1.25, *P* = 0.27; saltier site: *F*_1,68_ = 0.33, *P* = 0.57) or for *S. acutus* at the fresher site (*F*_1,68_ = 1.69, *P* = 0.20). There was a significant relationship for *S. acutus* at the saltier site; however, initial biomass explained very little variation in the final total biomass (*F*_1,67_ = 4.56, *P* = 0.036; *R* ^2^ = 0.05).

### 3.3 Abiotic effects on biomass

**Figure 2:**
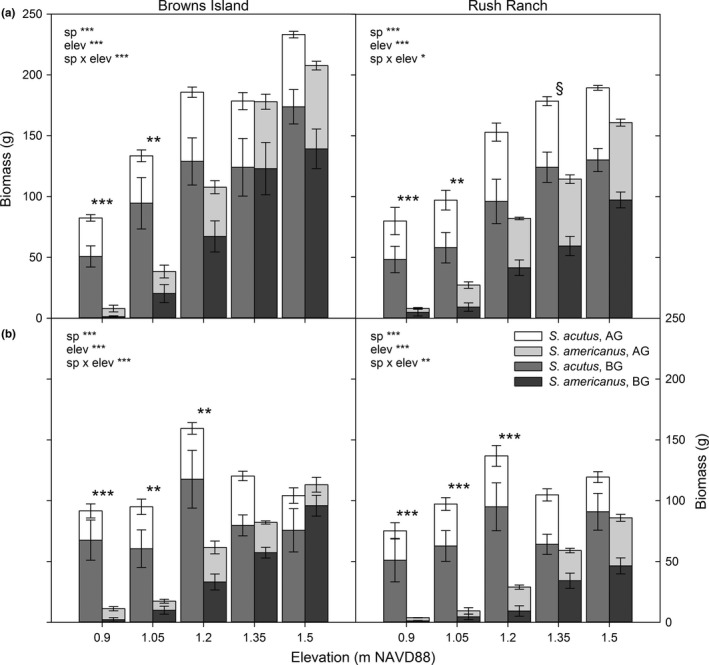
Aboveground (AG), belowground (BG), and total biomass of *S. acutus* and *S. americanus* grown a) alone and b) together at different elevations at the fresher, Browns Island, and saltier, Rush Ranch, sites (*N* = 7; error bars = ±1 *SE*; ANOVA summary statistics are in the upper corner; ***P <* *0.0001, **P <* *0.001, and *P <* *0.05; ^§^significant differences between species for BG biomass only at *P *<* *0.05)

When grown alone, inundation reduced biomass of *S. americanus* more than *S. acutus* at both sites (fresher site: *F*_9,70_ = 7.37, *P *<* *0.0001; saltier site: *F*_9,69_ = 3.00, *P* = 0.03; *P *<* *0.03 for all Tukey's comparisons; Figure 2a). Regardless of salinity, total biomass of *S. americanus* decreased significantly with increasing inundation (*P *<* *0.03) except that the top two and subsequent lower two elevations did not differ significantly (*P *>* *0.2). At the fresher site, total biomass of *S. acutus* at the lowest elevation was significantly less than its biomass at all other elevations (*P *<* *0.03); otherwise, the effect was negligible (*P *>* *0.7). At the saltier site, *S. acutus* biomass did not differ across the top three or bottom three elevations (*P *>* *0.4), but biomass was greater in the top two elevations than in the bottom two (*P *<* *0.02). Similar effects of inundation were detected for both above‐ and belowground biomass, individually, and with average stem density and total stem length (Table S3, Figs S6 and S7). The end of season live‐to‐dead biomass ratio was not different across elevations or between species at the fresher site; however, the ratio increased significantly with increasing elevation and was greater for *S. acutus* than for *S. americanus* (Table S4, Fig. S8a). The root‐to‐shoot ratio was lower for *S. americanus* than for *S. acutus* at the fresher site but not at the saltier site and tended to be lower at the lower elevations compared to the highest two at both sites (Table S4; Fig. S9a).

When grown together, inundation effects were similar to those when grown alone; *S. americanus* had a greater reduction in biomass than *S. acutus* with increased inundation at both sites (fresher site: *F*_9,67_ = 8.53, *P *<* *0.0001; saltier site: *F*_9,69_ = 3.96, *P* = 0.006; Figure 2b). Total biomass of *S. americanus* was significantly lower than *S. acutus* at the lowest three elevations within each site (*P *<* *0.001; Figure 2b). At both sites, total biomass of *S. americanus* at each elevation was significantly greater than biomass in the adjacent lower elevation (*P *<* *0.02). Total biomass of *S. acutus* did not differ significantly across elevations within either site when grown together (*P *>* *0.2). Similar inundation effects were observed with above‐ and belowground biomass, individually, and with total stem length and average stem density (Table S3, Figs S6 and S7). The end of season live‐to‐dead biomass ratio was greater for *S. acutus* than for *S. americanus* at both sites and tended to increase with increasing elevation at both sites, although the pattern was not strong (Table S4, Fig. S8b). There were no differences detected for root‐to‐shoot ratio between species or across elevations at either site (Table S4; Fig. S9b).

### 3.4 Biotic effects on biomass

**Figure 3:**
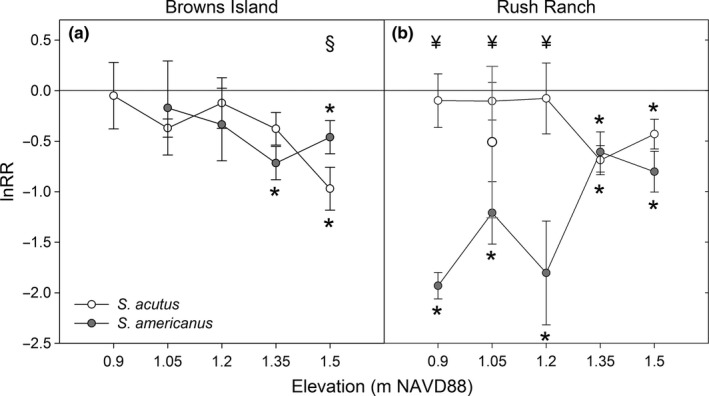
ln response ratio (lnRR) for the effect of biotic interactions on total biomass of *S. acutus* and *S. americanus* at a) the fresher site, Browns Island, and b) saltier site, Rush Ranch (error bars = ±1 *SE*). Individual values for each species are significantly different from 0 at *P <* *0.05. At ^¥^ *P *<* *0.05 and ^§^ *P *<* *0.08; species are significantly different from each other at a given elevation where noted. The individual circle at an elevation of 1.05 m at Rush Ranch denotes the average lnRR value with the influential replicate included

The presence of neighbors reduced total biomass of both species, particularly at the highest elevation; biomass did not increase with the presence of neighbors at any elevation across either site (Figures 2b and 3; Table S5). *Schoenoplectus acutus* was affected more negatively by the presence of its congener at the highest elevation at the fresher site compared to any other site/elevation combination (Figure 3; Table S5). Although the lnRR for *S. acutus* was significantly lower than zero (indicating a reduction in biomass and competitive effects) at the top two elevations at the saltier site, it never was outcompeted by *S. americanus* at any elevation (Figure 3; Table S5). Additionally, the lnRR for *S. americanus* was significantly lower than zero for all but one elevation and was affected more negatively by competition compared to its congener at the lowest three elevations (Figure 3; Table S5); biomass of *S. americanus* at the fresher site was reduced only at the top two elevations. The average lnRR for *S. americanus* at the middle elevation at the saltier site was influenced strongly by one replicate where plant performance was exceptionally great in the presence of *S. acutus* compared to that when grown alone (Figure 3). The replicate was not found to be an outlier (Grubb's test for outliers, *G* = 1.68 standard deviations from the mean). However, when it was removed from the analysis, a significant negative effect of competition was detected (*t*_2_ = −4.97, *P* = 0.04), and *S. americanus* performed worse than its congener (*F*_1,7_ = 8.34; *P* = 0.02; Figure 3).

## 4 Discussion

### 4.1 Direct effects of abiotic factors on growth

Our first objective was to document individual species’ responses to simulated SLR under field conditions. This experiment instantaneously increased inundation depths between 0.2 and 0.9 m relative to current average plant elevations, depths that are within the lower range of 2100 predictions of 0.4–1.8 m increases (Vermeer & Rahmstorf, 2009). *Schoenoplectus acutus*, the hypothesized stress tolerator, performed better under increased inundation stress than *S. americanus* (“better competitor”), supporting our first hypothesis. The general trend was consistent between wetlands with different salinity regimes, but the magnitude of biomass reduction for *S. americanus* was greater at the saltier site. Regardless of the presence of its congener and salinity, the low marsh species, *S. acutus*, tolerated greater inundation, growing at elevations 80 cm lower than its current average marsh distribution. Biomass of the marsh plain species, *S. americanus*, was greatly reduced when grown with increased inundation and decreased even more when grown with *S. acutus*; surviving plants had only 7% of the biomass at the lowest elevation compared to the highest elevation where it had the highest biomass (Figure 2). These findings are comparable to other studies that investigated the response of *S. americanus* to abiotic stress (Broome et al., 1995; Kirwan & Guntenspergen, 2012; Seliskar, 1990). Published data are limited on *S. acutus*; however, Sloey et al. (2016) measured a marked reduction in survival of *S. acutus* when inundated 100% of the time in a greenhouse experiment and documented a significant increase in cross‐sectional aerenchyma area with increased inundation. Additionally, Sloey et al. (2015) documented greater survival of *S. acutus* when rhizomes were transplanted with shoots, likely due to increased soil aeration. Biomass of both species was greater at the highest elevation tested when grown alone, suggesting that both species prefer growing under conditions of lower inundation. Despite a reduction in biomass at the lowest elevations, both species still displayed a remarkably broad tolerance to inundation, with survival of plants at an average inundation duration of up to 8 hr (Fig. S3). The elevation range in the experimental marsh organs was greater than the observed distribution of either species at both sites, which supports the short‐term resilience of both species to predicted increases in SLR. However, longer duration experiments are needed to assess whether these species will survive increased inundation over multiple years.

We documented a decrease in biomass in both species at the site with slightly higher channel water and pore water salinities (Table S2; Fig. S4), even though the difference in channel water salinity was only 3‰. Although we cannot say this conclusively due to the absence of replicate sites within each salinity range, we likely can attribute the reduced biomass to increased salinity because other environmental factors largely did not differ significantly between sites (Table S2). The depth of inundation was greater at the saltier site (Table S1), but the inundation duration was greater at the fresher site (Fig. S3), which we would argue has a stronger influence of soil biogeochemical processes and plant response (Casanova & Brock, 2000; Pezeshki, 2001). Considering this, biomass was higher at this site despite the increased inundation experienced. Water salinity in the San Francisco Bay estuary, as with other estuaries, is variable among years and with freshwater management practices. Growing season salinity is likely to increase into the future with increased sea level and reduced summer inflows; concentrations bay‐wide are predicted to increase between 3 and 5‰ by 2100 (Cloern et al., 2011).

### 4.2 Importance of biotic interactions along stress gradients

We predicted that the marsh plain species, *S. americanus*, would have greater biomass and competitive ability at higher elevations due to its rhizome morphology and overall marsh dominance (hypothesis 2); our data support this hypothesis but only under the most benign conditions tested: the top elevation at the fresher site (Figures 2b and 3). Within the experiment, *S. americanus* was grown at inundation levels that were much greater than at its current marsh distribution; therefore, any competitive advantage observed within its normal tidal elevations could have been overwhelmed by inundation stress. Under the two highest inundation levels at the fresher site, *S. americanus* had a reduction in biomass with little observed effect of competitive or facilitative interactions (Figure 3). Greiner La Peyre, Grace, Hahn, and Mendelssohn (2001) documented a similar pattern of reduced biomass for fresh and brackish marsh plants with increased stress (although with salinity rather than inundation stress). Inundation stress in this experiment was greater than what the plants normally experience in their current distributions and indicates a more negative effect on plant survival with predicted SLR that has not been documented previously.

An unexpected result not predicted by the stress gradient hypothesis was that, at the saltier site, *S. americanus* was competitively inferior to the stress tolerator, *S. acutus*, across most inundation levels (Figure 3). Furthermore, there was no evidence for facilitation under any treatment, and the negative effect of competition was amplified with increased inundation. Soil redox potential did not differ significantly across elevations (Fig. S5b) suggesting no detectible change in root soil aeration, which could have ameliorated stress from soil anaerobicity (Hacker & Bertness, 1999). A meta‐analysis by He et al. (2013) on the stress gradient hypothesis found a general shift toward facilitation with increased stress in coastal wetlands, with greater facilitation at high stress and neutral response at low stress in Mediterranean‐type environments similar to coastal California. In our experiment, however, not only was facilitation not documented with greater inundation, but the effect of competition was greater with increased inundation stress. This result is contrary to what we predicted (hypothesis 3): facilitation, which has been shown to increase with increased abiotic stress in salt, brackish, and freshwater marshes in other regions (Bertness & Callaway, 1994; Guo & Pennings, 2012; Halpern, Silliman, Olden, Bruno, & Bertness, 2007; Luo, Xie, Che, Li, & Qin, 2010), was not detected at any elevation.

### 4.3 Implications with climate change

As suggested by our data, predicting climate change effects likely will not be as straightforward as offered by the stress gradient hypothesis. The direct effects are clear and followed what was predicted based on existing distributions/tolerances; however, the indirect effects of climate change on species interactions are more complicated and not predictable. In our case, we documented no facilitation by the stress tolerator under the greatest simulated SLR and demonstrated that its presence was more deleterious for the other species. Regardless of species interactions, we documented that the low marsh species could grow at elevations that were 80 cm lower than its current average elevation, which indicates its high inundation tolerance and potential to persist under conditions predicted by increased sea levels. Although species diversity may be reduced across the marsh as a whole, our results suggest that these wetlands in theory could remain vegetated in light of increased submergence.

Multifactor manipulation experiments addressing plant response to accelerated climate change are uncommon and often are expensive to run, yet provide a more realistic, valuable, and needed assessment of how systems might respond (Dieleman et al., 2012; Langley & Megonigal, 2010; Rustad, 2008). Oftentimes, addressing factors individually produces results that vary significantly than in combined treatments and can influence model results (Dieleman et al., 2012). Our results point to the importance of synergies between multiple stressors, which are predicted to increase in intensity with climate change, as well as the consideration of species interactions. When species are exposed to the stressor that ultimately is limiting at the edge of its range, differential effects of biotic interactions might occur (Guo & Pennings, 2012; Maestre et al., 2009). We did not demonstrate shifts in the nature of biotic interactions in this study and found that increased inundation, salinity, and competition compromised the ability of both species, particularly the marsh plain species, to grow.

The implications of reduced biomass and lack of facilitative effects for marsh sustainability under increased SLR are significant. We observed a reduction in biomass for both species examined that was amplified with an increase in salinity of just 3‰. This reduction implies that there will be a decrease in the organic matter contribution to marsh accretion that could compound the loss of elevation and inundation stress within the marsh, especially for freshwater and brackish marshes that have organic rich soils (Craft, 2007; Callaway et al. 2012). Wetlands respond to increases in sea level through increased sediment deposition (Morris et al., 2002) and plant growth (Cherry et al., 2009; Kirwan & Megonigal, 2013; Langley, McKee, Cahoon, Cherry, & Megonigal, 2009) to maintain their elevation. These factors, combined with upland migration, would reduce the negative impact of increasing sea levels. However, there are limits to these responses (Kirwan, Guntenspergen, D'Alpaos, & Morris, 2010), and once low marsh species drop below the elevation of peak biomass, the marsh is likely to continue losing elevation (Morris et al., 2002). Growing evidence suggests that a reduction in suspended sediment concentrations (Cloern et al., 2011; Ibàñez, Prat, & Canicio, 1996), reduced biomass due to individual plant responses and competitive interactions (this study), and a limited amount of available upland habitat (Schile et al., 2014; Stralberg et al., 2011) present a future of shrinking tidal wetland extent.

## Conflict of Interest

None declared.
