# Supplementary material for: Can community structure track sea‐level rise? Stress and competitive controls in tidal wetlands
Source: Ecol Evol. 2017 Jan 27;7(4):1276–85. doi: 10.1002/ece3.2758 (PMC5305999; doi:10.1002/ece3.2758)
Supplement: Supplementary file 1 [file ECE3-7-1276-s001.docx]

**Supporting Information**

Table S1. Tidal metrics in meters NAVD88 at the study sites (MHHW = mean higher high water; MHW = mean high water; MLHW = mean lower high water; MTL = mean tide level; MHLW = mean higher low water; MLW = mean low water; MLLW = mean lower low water).

|  |  |  |  |  |  |  |  |
| --- | --- | --- | --- | --- | --- | --- | --- |
| Site | MHHW | MHW | MLHW | MTL | MHLW | MLW | MLLW |
| Browns Is. | 1.90 | 1.76 | 1.62 | 1.31 | 1.03 | 0.84 | 0.63 |
| Rush Ranch | 2.01 | 1.87 | 1.73 | 1.29 | 0.93 | 0.69 | 0.46 |

Table S2. Results of a two-way ANOVA (inundation duration) and rmANOVA (salinity, sulfides, and *Eh*) on the effects of model (M), time (T), site (S), elevation (E), and their interaction on abiotic measurements.

|  |  | *df* | *F* | *P* |
| --- | --- | --- | --- | --- |
| *Inundation duration* | |  |  |  |
|  | M | 9, 2510 | 224.54 | <0.0001 |
|  | S | 1, 2510 | 53.71 | <0.0001 |
|  | E | 4, 2510 | 477.98 | <0.0001 |
|  | S × E | 4, 2510 | 8.19 | <0.0001 |
| *Salinity* |  |  |  |  |
|  | T | 4, 52 | 408.71 | <0.0001 |
|  | S | 4, 52 | 70.84 | <0.0001 |
|  | E | 16, 160 | 3.87 | <0.0001 |
|  | T × S × E | 16, 160 | 0.58 | 0.89 |
| *Sulfides* |  |  |  |  |
|  | T | 4, 56 | 4.73 | 0.002 |
|  | S | 4, 56 | 1.97 | 0.11 |
|  | E | 16, 172 | 0.99 | 0.47 |
|  | T × S × E | 16, 172 | 0.58 | 0.63 |
| *Eh* |  |  |  |  |
|  | T | 4, 9 | 5.65 | 0.015 |
|  | S | 4, 9 | 13.95 | 0.0007 |
|  | E | 16, 28.1 | 1.23 | 0.31 |
|  | T × S × E | 16, 28.1 | 2.10 | 0.04 |

Table S3. Results of two-way ANOVA testing the effects of model (M), species (S), elevation (E), and their interaction on plant productivity metrics.

|  |  |  |  | |  |  |  |  | |  |  |  |  | |  |  |  |  | |  |
| --- | --- | --- | --- | --- | --- | --- | --- | --- | --- | --- | --- | --- | --- | --- | --- | --- | --- | --- | --- | --- |
|  |  | *Browns Island* | | | | | | | | |  | *Rush Ranch* | | | | | | | | |
|  |  | alone | | | |  | competition | | | |  | Alone | | | |  | competition | | | |
|  |  | *df* | | *F* | *P* |  | *df* | | *F* | *P* |  | *df* | | *F* | *P* |  | *df* | | *F* | *P* |
| *Total biomass* | |  | |  |  |  |  | |  |  |  |  | |  |  |  |  | |  |  |
|  | M | 9, 70 | | 26.81 | <0.0001 |  | 9, 67 | | 15.80 | <0.0001 |  | 9, 69 | | 20.86 | <0.0001 |  | 9, 69 | | 17.69 | <0.0001 |
|  | S | 1, 70 | | 44.30 | <0.0001 |  | 1, 67 | | 48.72 | <0.0001 |  | 1, 69 | | 65.92 | <0.0001 |  | 1, 69 | | 108.02 | <0.0001 |
|  | E | 4, 70 | | 42.23 | <0.0001 |  | 4, 67 | | 13.76 | <0.0001 |  | 4, 69 | | 27.45 | <0.0001 |  | 4, 69 | | 8.84 | <0.0001 |
|  | S × E | 4, 70 | | 7.37 | <0.0001 |  | 4, 67 | | 8.53 | <0.0001 |  | 4, 69 | | 3.00 | 0.02 |  | 4, 69 | | 3.96 | 0.006 |
| *Aboveground biomass* | |  | |  |  |  |  | |  |  |  |  | |  |  |  |  | |  |  |
|  | M | 9, 70 | | 25.35 | <0.0001 |  | 9, 67 | | 11.19 | <0.0001 |  | 9, 69 | | 14.60 | <0.0001 |  | 9, 69 | | 15.34 | <0.0001 |
|  | S | 1, 70 | | 33.13 | <0.0001 |  | 1, 67 | | 34.42 | <0.0001 |  | 1, 69 | | 54.61 | <0.0001 |  | 1, 69 | | 107.46 | <0.0001 |
|  | E | 4, 70 | | 38.10 | <0.0001 |  | 4, 67 | | 10.68 | <0.0001 |  | 4, 69 | | 14.59 | <0.0001 |  | 4, 69 | | 4.86 | 0.002 |
|  | S × E | 4, 70 | | 10.78 | <0.0001 |  | 4, 67 | | 5.15 | 0.001 |  | 4, 69 | | 4.62 | 0.003 |  | 4, 69 | | 2.80 | 0.03 |
| *Belowground biomass* | |  | |  |  |  |  | |  |  |  |  | |  |  |  |  | |  |  |
|  | M | 9, 70 | | 22.06 | <0.0001 |  | 9, 67 | | 14.68 | <0.0001 |  | 9, 69 | | 22.31 | <0.0001 |  | 9, 69 | | 18.08 | <0.0001 |
|  | S | 1, 70 | | 39.28 | <0.0001 |  | 1, 67 | | 42.77 | <0.0001 |  | 1, 69 | | 64.08 | <0.0001 |  | 1, 69 | | 103.38 | <0.0001 |
|  | E | 4, 70 | | 35.15 | <0.0001 |  | 4, 67 | | 13.22 | <0.0001 |  | 4, 69 | | 32.11 | <0.0001 |  | 4, 69 | | 10.52 | <0.0001 |
|  | S × E | 4, 70 | | 5.04 | 0.001 |  | 4, 67 | | 8.18 | <0.0001 |  | 4, 69 | | 2.06 | 0.09 |  | 4, 69 | | 4.31 | 0.004 |

Table S4. Results of two-way ANOVA testing the effects of model (M), species (S), elevation (E), and their interaction on live to dead biomass and root to shoot ratios.

|  |  |  |  | |  |  |  |  | |  |  |  |  | |  |  |  |  | |  |
| --- | --- | --- | --- | --- | --- | --- | --- | --- | --- | --- | --- | --- | --- | --- | --- | --- | --- | --- | --- | --- |
|  |  | *Browns Island* | | | | | | | | |  | *Rush Ranch* | | | | | | | | |
|  |  | alone | | | |  | competition | | | |  | Alone | | | |  | competition | | | |
|  |  | *df* | | *F* | *P* |  | *df* | | *F* | *P* |  | *df* | | *F* | *P* |  | *df* | | *F* | *P* |
| *Live:dead biomass ratio* | |  | |  |  |  |  | |  |  |  |  | |  |  |  |  | |  |  |
|  | M | 9, 55 | | 1.83 | 0.0825 |  | 9, 50 | | 2.83 | 0.0090 |  | 9, 54 | | 5.86 | <0.0001 |  | 9, 48 | | 5.07 | <0.0001 |
|  | S | 1, 55 | | 2.41 | 0.1259 |  | 1, 50 | | 9.05 | 0.0041 |  | 1, 54 | | 36.73 | <0.0001 |  | 1, 48 | | 30.95 | <0.0001 |
|  | E | 4, 55 | | 1.67 | 0.1695 |  | 4, 50 | | 3.85 | 0.0084 |  | 4, 54 | | 4.53 | 0.0032 |  | 4, 48 | | 4.59 | 0.0032 |
|  | S × E | 4, 55 | | 1.34 | 0.2652 |  | 4, 50 | | 0.81 | 0.5219 |  | 4, 54 | | 0.55 | 0.7005 |  | 4, 48 | | 0.88 | 0.4830 |
| *Root:shoot ratio* | |  | |  |  |  |  | |  |  |  |  | |  |  |  |  | |  |  |
|  | M | 9, 56 | | 2.93 | 0.0064 |  | 9, 48 | | 0.92 | 0.5188 |  | 9, 53 | | 3.37 | 0.0025 |  | 9, 51 | | 0.98 | 0.4683 |
|  | S | 1, 56 | | 8.17 | 0.0060 |  | 1, 48 | | 0.00 | 0.9525 |  | 1, 53 | | 0.10 | 0.7506 |  |  | |  |  |
|  | E | 4, 56 | | 4.09 | 0.0056 |  | 4, 48 | | 1.05 | 0.3907 |  | 4, 53 | | 4.71 | 0.0025 |  |  | |  |  |
|  | S × E | 4, 56 | | 1.13 | 0.3513 |  | 4, 48 | | 0.91 | 0.4671 |  | 4, 53 | | 2.46 | 0.0561 |  |  | |  |  |

Table S5. Results of the lnRR test for the direction and strength of biotic interactions and species differences on total plant biomass when grown at Browns Island (B) and Rush Ranch (R) across five elevations. (* see results about removal of outlier)

|  |  |  |  |  |  |  |  |  |  |  |  |  |
| --- | --- | --- | --- | --- | --- | --- | --- | --- | --- | --- | --- | --- |
|  |  | *S. acutus* | | |  | *S. americanus* | | |  | Species comparison | | |
| Site | elevation (m) | *df* | *t-value* | *P* |  | *df* | *t-value* | *P* |  | *df* | *F* | *P* |
| B | 0.90 | 6 | 0.62 | 0.56 |  | 1 | - | - |  | 1, 6 | 1.32 | 0.29 |
| B | 1.05 | 6 | -2.34 | 0.05 |  | 4 | -0.37 | 0.73 |  | 1, 10 | 0.8 | 0.39 |
| B | 1.20 | 6 | -0.49 | 0.64 |  | 5 | -0.93 | 0.39 |  | 1, 11 | 0.25 | 0.63 |
| B | 1.35 | 6 | -2.34 | 0.05 |  | 6 | -4.4 | 0.005 |  | 1, 12 | 2.2 | 0.16 |
| B | 1.50 | 5 | -4.58 | 0.006 |  | 5 | -2.82 | 0.04 |  | 1, 10 | 2.7 | 0.08 |
| R | 0.90 | 6 | -0.37 | 0.72 |  | 1 | -14.93 | 0.04 |  | 1, 7 | 12.18 | 0.01 |
| R | 1.05 | 5 | -0.56 | 0.60 |  | 3* | -0.68 | 0.55 |  | 1, 8* | 0.4 | 0.54 |
| R | 1.20 | 6 | -0.22 | 0.83 |  | 5 | -3.52 | 0.02 |  | 1, 11 | 8.12 | 0.02 |
| R | 1.35 | 6 | -4.76 | 0.003 |  | 6 | -3.06 | 0.02 |  | 1, 12 | 0.11 | 0.75 |
| R | 1.50 | 6 | -2.92 | 0.03 |  | 6 | -4 | 0.007 |  | 1, 12 | 2.23 | 0.16 |





**Fig. S1.** Study sites in the San Francisco Bay Estuary, California, USA.


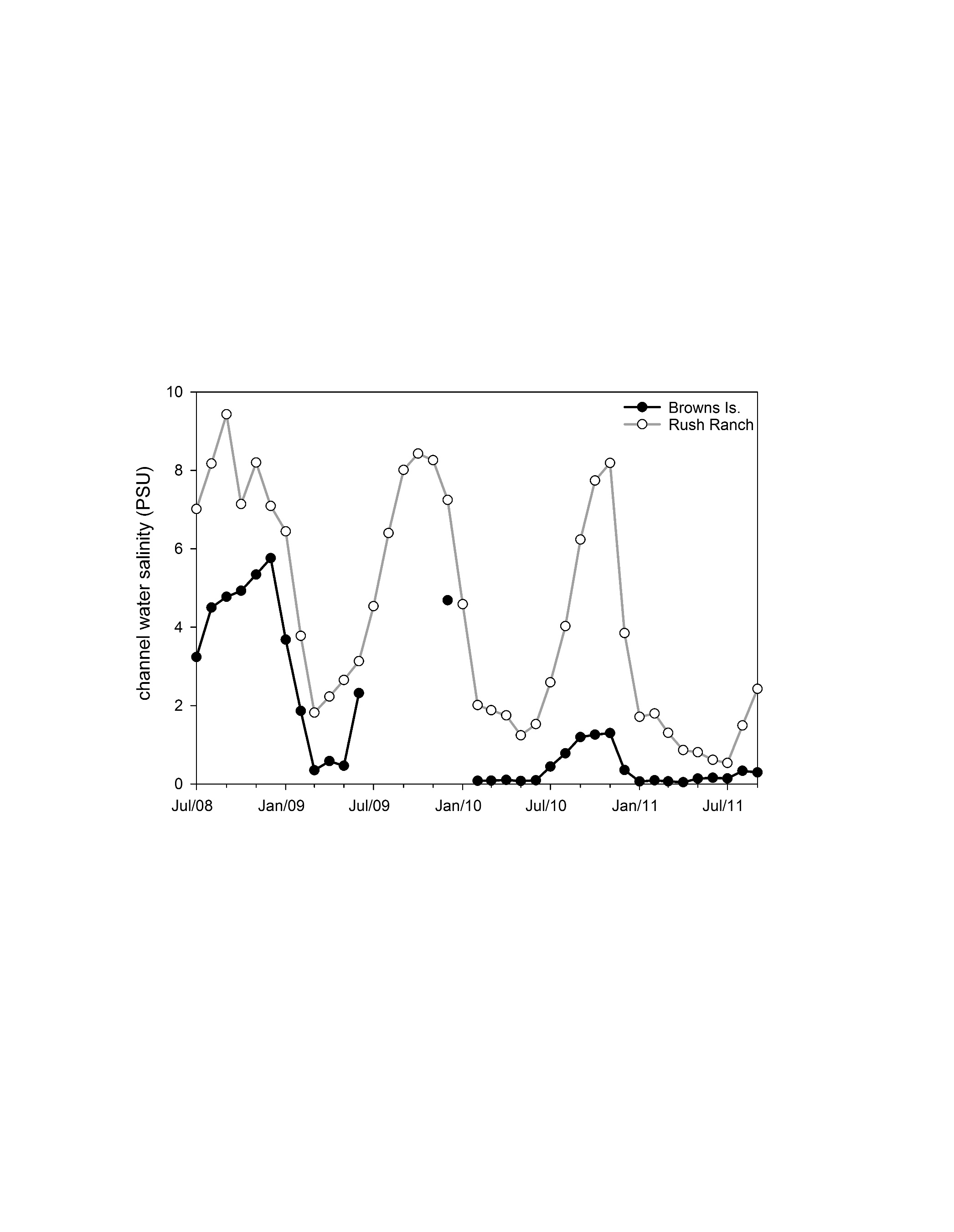


**Fig. S2.** Average monthly channel water salinity at Browns Island and Rush Ranch.


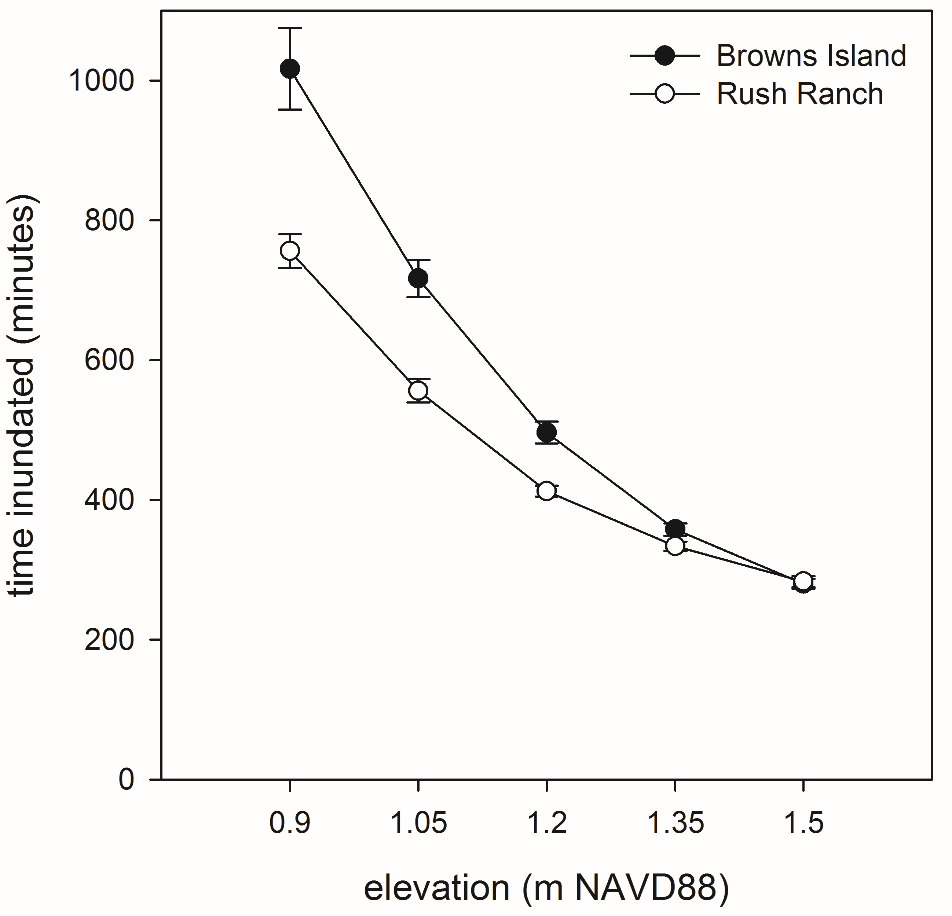


**Fig. S3.** Average inundation time per tide cycle at each elevation treatment at Browns Island and Rush Ranch (error bars = ±1 SE).


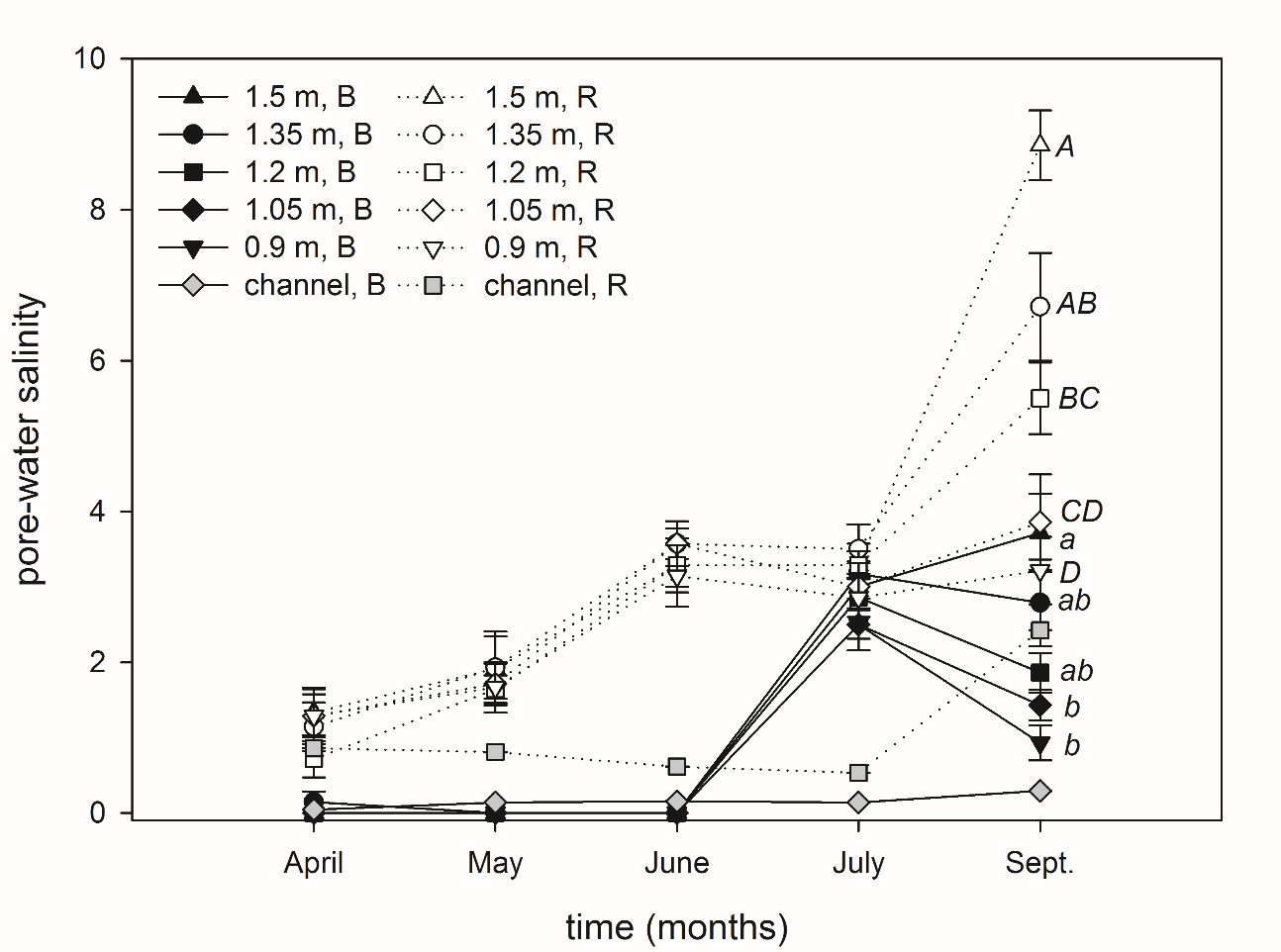


**Fig. S4.** Pore-water and channel water salinity at Rush Ranch (R) and Browns Island (B) at each elevation treatment over time (error bars = ±1 SE). Capital and lower-case letters denote significant differences at *P* < 0.05 across elevations in September at Rush Ranch and Browns Island, respectively.


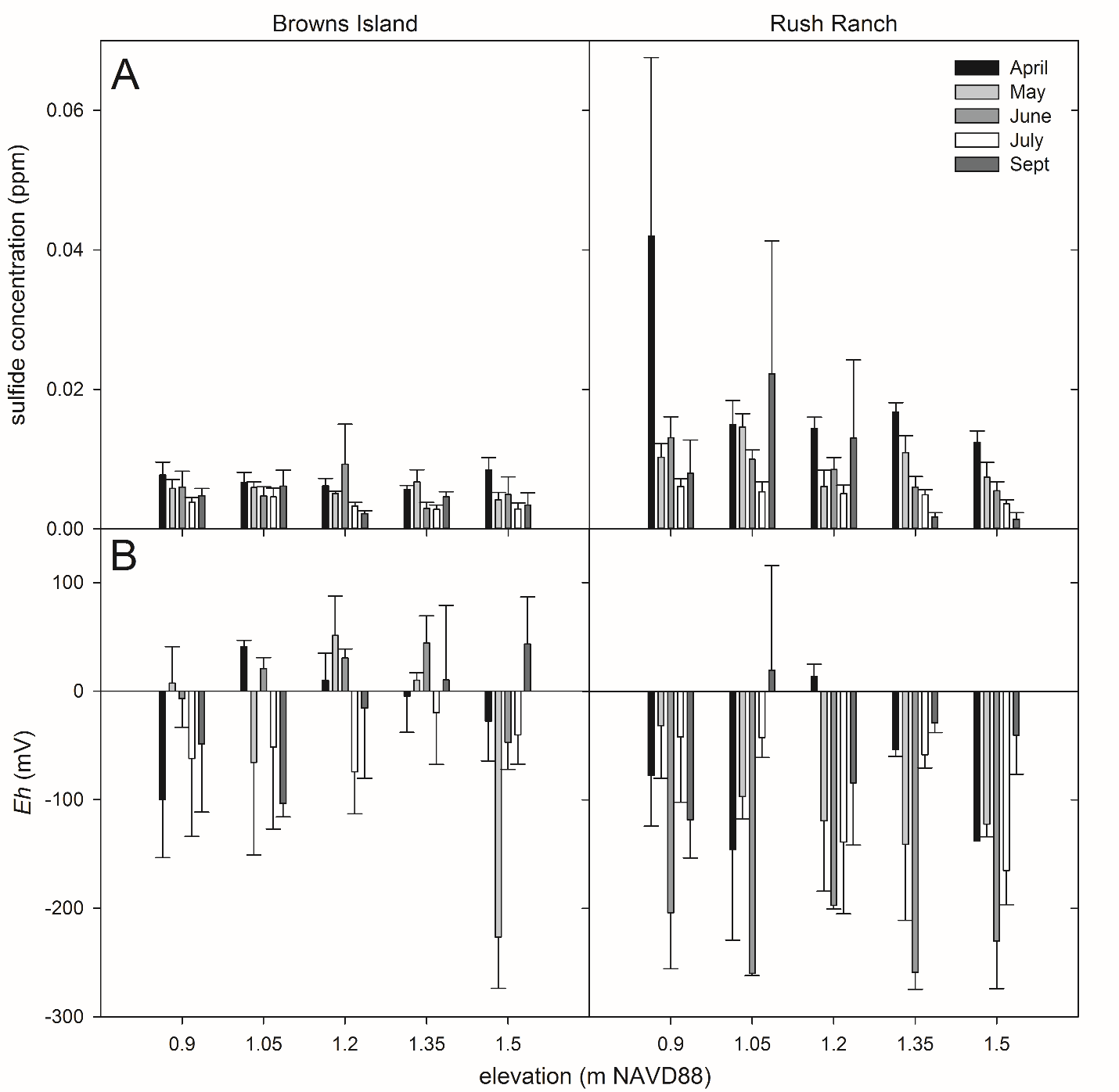


**Fig. S5.** A) Sulfide concentrations and B) redox potential (*Eh*) at Browns Island and Rush Ranch over time at each elevation treatment (error bars = ±1 SE).


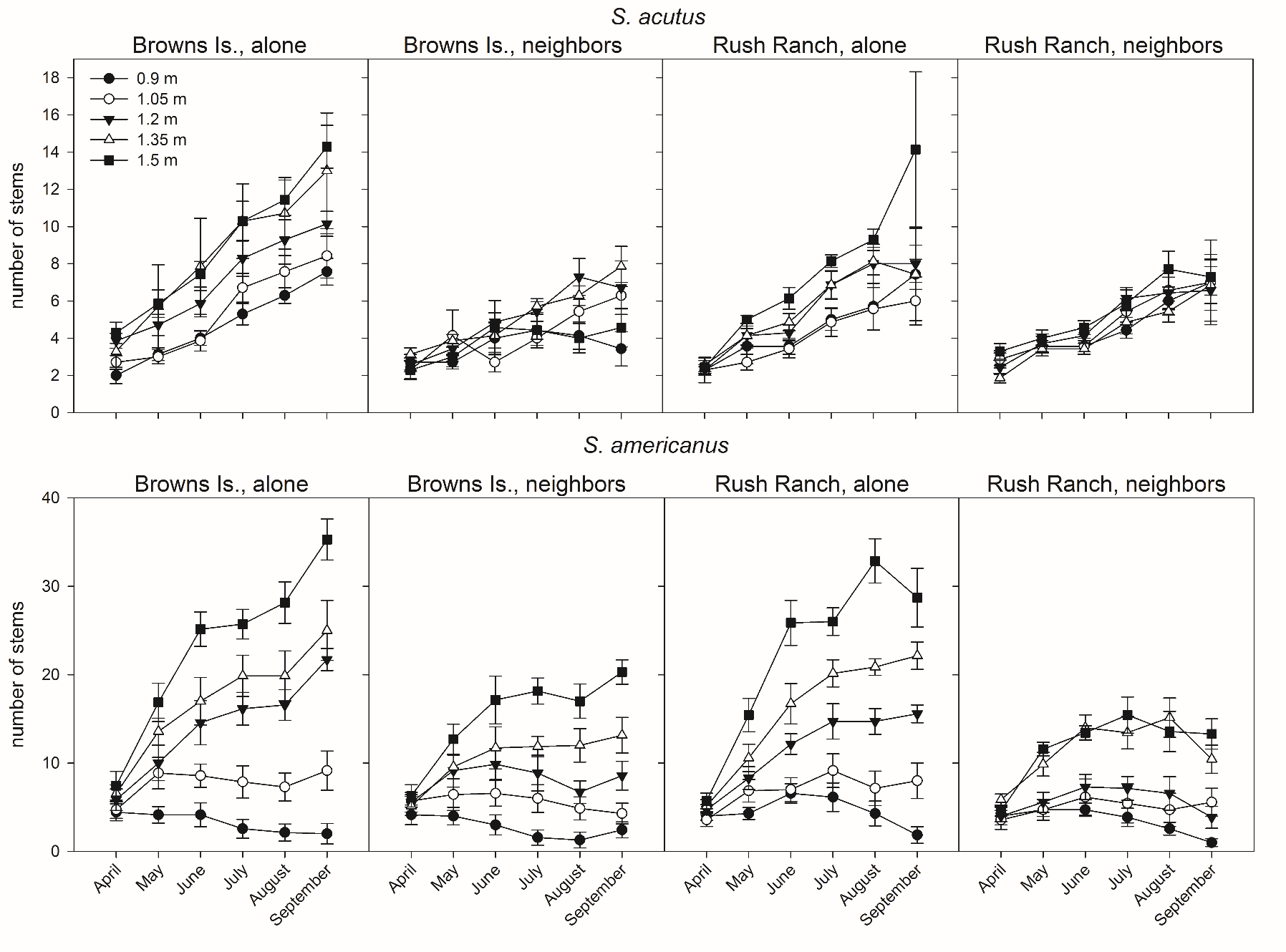


**Fig. S6.** Average number of plant stems per marsh organ pipe at each elevation and species treatment (error bars = ±1 SE).


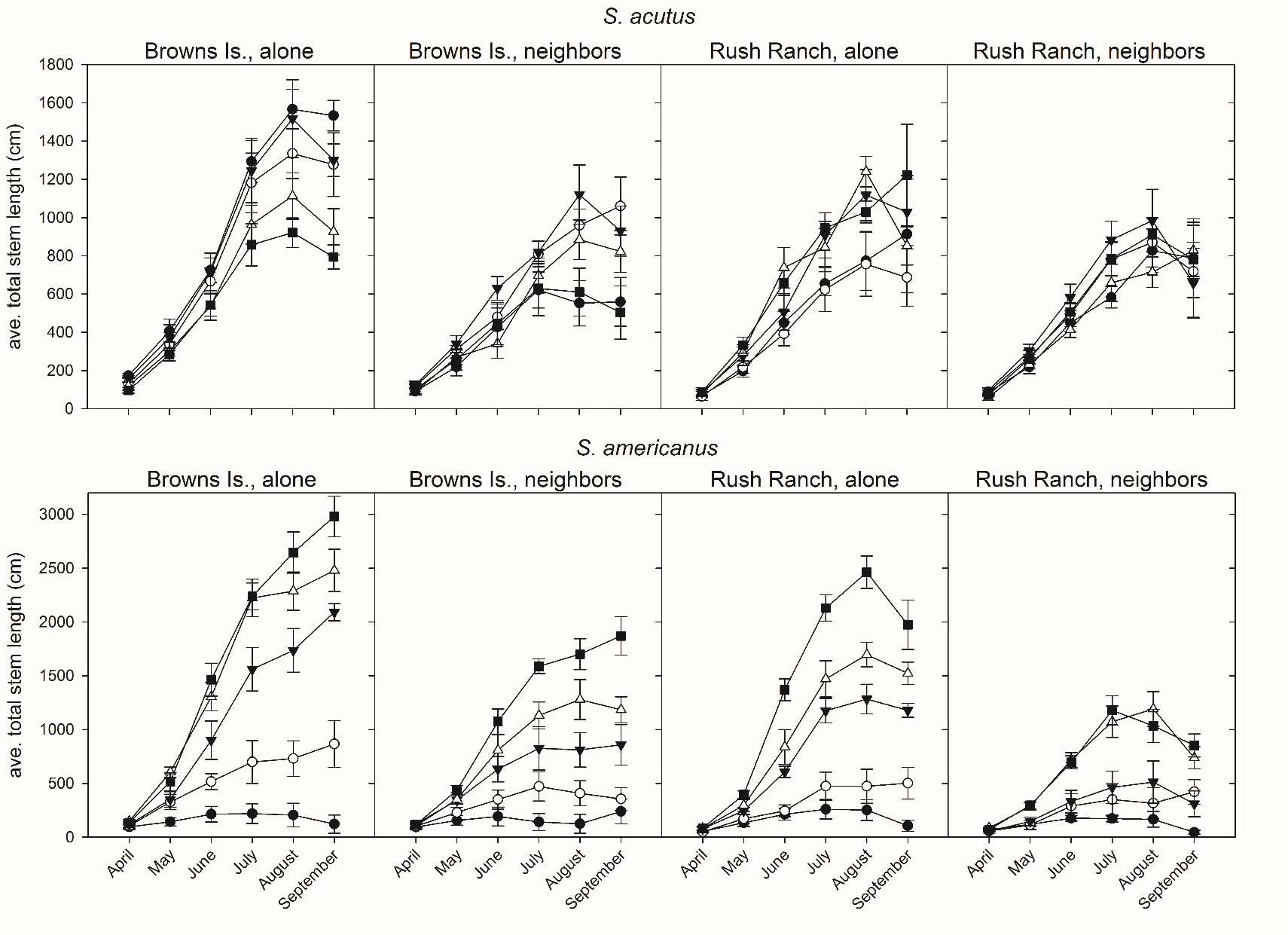


**Fig. S7.** Average total stem length per marsh organ pipe at each elevation and species treatment (error bars = ±1 SE).


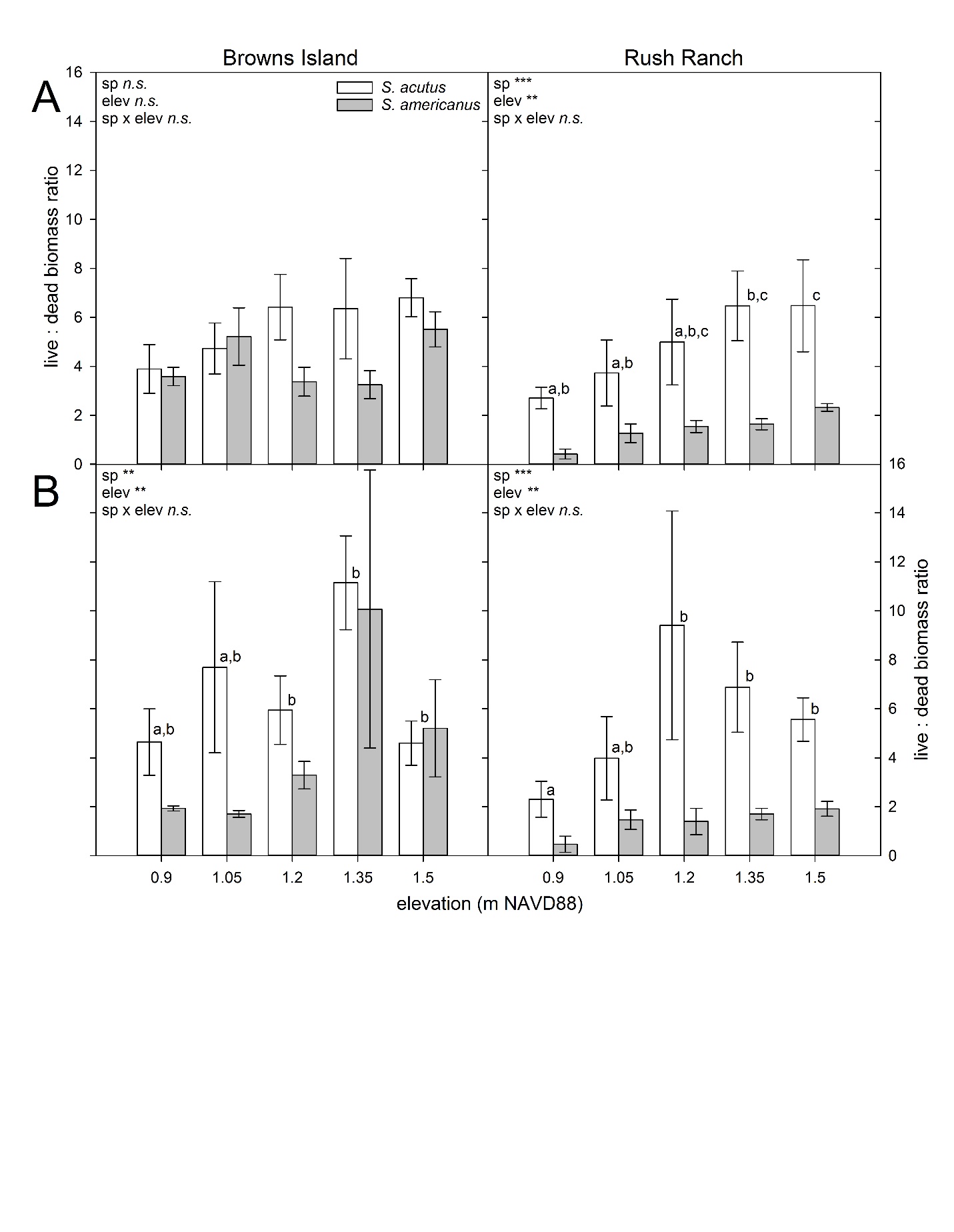


**Fig S8.** End-of-season live to dead biomass ratio of *S. acutus* and *S. americanus* when grown a) individually and b) together across elevations at each site (N = 7; error bars = ±1 SE; ANOVA summary statistics are in the upper corner; *** < 0.0001, ** = *P* < 0.01, and *n.s.* = not significant; when there is a significant elevation effect, letters denote significant differences across elevations at *P* < 0.05).


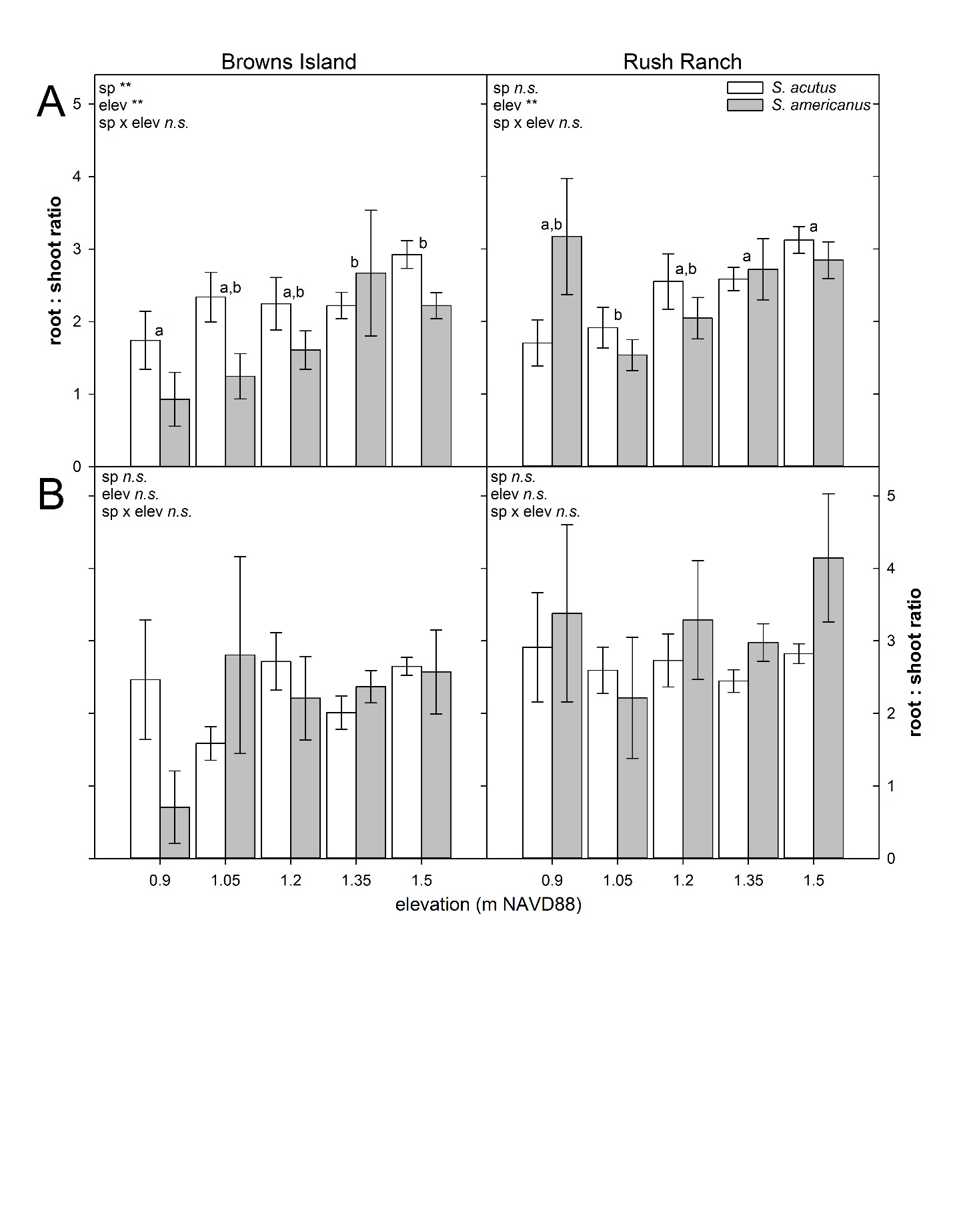


**Fig. S9.** Root to shoot ratios of *S. acutus* and *S. americanus* when grown a) individually and b) together across elevations at each site (N = 7; error bars = ±1 SE; ANOVA summary statistics are in the upper corner; ** = *P* < 0.001 and *n.s.* = not significant; for the significant elevation effect at Rush Ranch when species are grown individually, letters denote significant differences across elevations at *P* < 0.04).
